# Lung Nodules Missed in Initial Staging of Breast Cancer Patients in PET/MRI—Clinically Relevant?

**DOI:** 10.3390/cancers14143454

**Published:** 2022-07-15

**Authors:** Kai Jannusch, Nils Martin Bruckmann, Charlotte Johanna Geuting, Janna Morawitz, Frederic Dietzel, Christoph Rischpler, Ken Herrmann, Ann-Kathrin Bittner, Oliver Hoffmann, Svjetlana Mohrmann, Harald H. Quick, Lale Umutlu, Gerald Antoch, Julian Kirchner

**Affiliations:** 1 Department of Diagnostic and Interventional Radiology, Medical Faculty, University Dusseldorf, 40225 Dusseldorf, Germany; kai.jannusch@med.uni-duesseldorf.de (K.J.); nils-martin.bruckmann@med.uni-duesseldorf.de (N.M.B.); charlotte.geuting@gmx.de (C.J.G.); janna.morawitz@med.uni-duesseldorf.de (J.M.); frederic.dietzel@med.uni-duesseldorf.de (F.D.); antoch@med.uni-duesseldorf.de (G.A.); 2 Department of Nuclear Medicine, University Hospital Essen, University of Duisburg-Essen, 45147 Essen, Germany; christoph.rischpler@uk-essen.de (C.R.); ken.herrmann@uk-essen.de (K.H.); 3 Department Gynecology and Obstetrics, University Hospital Essen, University of Duisburg-Essen, 45147 Essen, Germany; ann-kathrin.bittner@uk-essen.de (A.-K.B.); oliver.hoffmann@uk-essen.de (O.H.); 4 Department of Gynecology, Medical Faculty, University Dusseldorf, 40225 Dusseldorf, Germany; mohrmann@med.uni-duesseldorf.de; 5 High-Field and Hybrid MR Imaging, University Hospital Essen, University Duisburg-Essen, 45147 Essen, Germany; harald.quick@uk-essen.de; 6 Erwin L. Hahn Institute for Magnetic Resonance Imaging, University Duisburg-Essen, 45141 Essen, Germany; 7 Department of Diagnostic and Interventional Radiology and Neuroradiology, University Hospital Essen, University of Duisburg-Essen, 45147 Essen, Germany; lale.umutlu@uk-essen.de

**Keywords:** PET/MRI, breast cancer, lung nodules, staging

## Abstract

**Simple Summary:**

Image-based primary staging in women with newly-diagnosed breast cancer is important to exclude distant metastases, which affect up to 10% of women. The increasing implementation of [^18^F]FDG-PET/MRI as a radiation-saving primary staging tool bears the risk of missing lung nodules. Thus, chest CT serves as the diagnostic of choice for the detection and classification of pulmonary nodules. The aim of this study was the evaluation of the clinical relevance of missed lung nodules at initial staging of breast cancer patients in [^18^F]FDG-PET/MRI compared with CT. We demonstrated in an homogeneous population of 152 patients that all patients with newly-diagnosed breast cancer and clinically-relevant lung nodules were detected at initial [^18^F]FDG-PET/MRI staging. However, due to the lower sensitivity of MRI in detecting lung nodules, a small proportion of clinically-relevant lung nodules were missed. Thus, a supplemental low-dose chest CT after neoadjuvant therapy should be considered for backup.

**Abstract:**

Purpose: The evaluation of the clinical relevance of missed lung nodules at initial staging of breast cancer patients in [^18^F]FDG-PET/MRI compared with CT. Methods: A total of 152 patients underwent an initial whole-body [^18^F]FDG-PET/MRI and a thoracoabdominal CT for staging. Presence, size, shape and location for each lung nodule in [^18^F]FDG-PET/MRI was noted. The reference standard was established by taking initial CT and follow-up imaging into account (a two-step approach) to identify clinically-relevant lung nodules. Patient-based and lesion-based data analysis was performed. Results: No patient with clinically-relevant lung nodules was missed on a patient-based analysis with MRI VIBE, while 1/84 females was missed with MRI HASTE (1%). Lesion-based analysis revealed 4/96 (4%, VIBE) and 8/138 (6%, HASTE) missed clinically-relevant lung nodules. The average size of missed lung nodules was 3.2 mm ± 1.2 mm (VIBE) and 3.6 mm ± 1.4 mm (HASTE) and the predominant location was in the left lower quadrant and close to the hilum. Conclusion: All patients with newly-diagnosed breast cancer and clinically-relevant lung nodules were detected at initial [^18^F]FDG-PET/MRI staging. However, due to the lower sensitivity in detecting lung nodules, a small proportion of clinically-relevant lung nodules were missed. Thus, supplemental low-dose chest CT after neoadjuvant therapy should be considered for backup.

## 1. Introduction

Breast cancer is the most common solid tumor in women worldwide, accounting for 11.7% of all cancers diagnoses and approximately 2.3 million new cases every year [1]. In addition to knowledge of tumor biology, accurate knowledge of the TNM stage is essential for therapy planning [2,3]. Therefore, imaging plays a key role in the diagnostic workup of breast cancer patients. Current guidelines recommend chest/abdomen computed tomography (CT) and bone scintigraphy for staging. Especially in unequivocal cases, ^18^fluorodeoxyglucose ([^18^F]FDG) Positron emission tomography (PET)/CT can be used as a supporting diagnostic tool [4,5]. Furthermore, some studies suggest performing initial staging with PET/CT in predefined patient groups [5,6,7]. As an alternative to PET/CT, PET/magnet resonance imaging (MRI) is gaining visibility as a staging method for breast cancer patients by combining high-quality whole-body staging and multiparametric breast MRI, and has shown its superiority over PET/CT, MRI and CT [8,9,10,11]. However, detecting small lung nodules remains a major problem in MRI, and CT is still the golden standard for lung evaluation. One reason for this is the susceptibility of MRI to respiratory and cardiac motion resulting in a markedly reduced accessibility of the lung parenchyma and limited detectability of potentially metastatic lung nodules [12]. Despite technical advances in MR imaging over the years, several studies showed that lung nodules, especially small ones, continue to be missed [12,13,14]. In general, interpreting small lung nodules represents a problem in radiological diagnostics. Even though the majority of these lung nodules are benign (i.e., post-infectious or indurative genesis), it is important to ensure that no malignant lung nodules are missed [15,16]. Thus, a clinically-balanced staging is necessary which, on the one hand, does not negate any relevant findings; but, on the other hand, does not lead to an unjustified upstaging or follow-up imaging. For this purpose, the current Fleischner Society recommendations provide clinically-accepted guidelines on how to deal with small pulmonary nodules by recommending follow-up examination for nodules > 6 mm at different time intervals, depending on size, morphology, and patients history [17].

The aim of this study was to evaluate the clinical relevance of missed lung nodules at initial staging of breast cancer patients in [^18^F]FDG-PET/MRI compared with CT.

## 2. Materials and Methods

### 2.1. Patients

The institutional review boards of the University Duisburg-Essen, Germany (study number 17-7396-BO) and Düsseldorf, Germany (study number 6040 R) approved this study, and it was performed in conformance with the Declaration of Helsinki [18]. Written informed consent form was obtained from all patients. Inclusion criteria were defined as follows: (1) newly diagnosed, treatment-naive T2 tumor or higher T-stage; or (2) newly diagnosed, treatment-naive triple-negative tumor of every size; or (3) newly diagnosed, treatment-naive tumor with molecular high risk (T1 c, Ki67 >  14%, HER2-new over-expression, G3). Exclusion criteria were contraindications to MRI or MRI contrast agents, missing imaging of a modality, pregnancy or breast-feeding and former malignancies in the last 5 years. Inclusion criteria were chosen according to clinical ESMO guidelines to set elevated pre-test probability for distant metastases [19,20]. Data acquisition was performed between March 2018 and December 2021.

A total of 152 women with newly-diagnosed breast cancer (mean age: 53 years ± 12 years; range 30–81 years) were included in this study. As part of this study all women underwent a whole-body [^18^F]FDG-PET/MRI examination for staging purposes in addition to a thoraco-abdominal CT with a maximum of 30 days apart (mean: 5.2 days ± 5.4 days). Due to cohort size, no further subgroup analyses was performed regarding different cancer subtypes.

### 2.2. PET/MRI

All [^18^F]FDG-PET/MRI examinations were performed on an integrated 3-Tesla PET/MRI system (Biograph mMR, Siemens Healthcare GmbH, Erlangen, Germany). Examinations were performed one hour after injection of bodyweight-adapted dosage of [^18^F]FDG (4 MBq/kg bodyweight). The imaging protocol included a dedicated breast [^18^F]FDG-PET/MRI and a whole-body imaging scan [21]. Only the whole-body PET/MR examinations were considered for this study. [^18^F]FDG-PET images were reconstructed after a standard protocol [22]. MRI data were acquired simultaneously using a 16-channel head-and-neck radiofrequency (RF) coil, a 24-channel spine array RF coil and 5 or 6-channel flex body coils, depending on patients’ height. The WB-MRI protocol comprised the following sequences:

i. A transverse T2-w half Fourier acquisition single shot turbo spin echo (HASTE) sequence in breath-hold technique with a slice thickness of 7 mm (TE 97 ms; TR 1500 ms; turbo factor (TF) 194; FOV 400 mm; phase FOV 75%; acquisition matrix 320 × 240 mm; in plane resolution 1.3 × 1.3 mm; TA 0:47 min/bed position)
ii. A fat-saturated post-contrast transverse 3-dimensional volumetric interpolated breath-hold examination (VIBE) sequence with a slice thickness of 3 mm (TE, 1.53 ms; TR, 3.64 ms; flip angle 9°; FOV 400 × 280 mm; phase FOV 75%; acquisition matrix 512 × 384, in-plane resolution 0.7 × 0.7 mm; TA 0:19 min/bed position)
iii. A transversal diffusion-weighted (DW) echo-planar imaging (EPI) sequence in free breathing with a slice thickness of 5.0 mm (TR 7400 ms; TE 72 ms; b-values: 0, 500, and 1000 s/mm^2^, matrix size 160 × 90; FOV 400 mm × 315 mm, phase FOV, 75%; GRAPPA, acceleration factor 2; in-plane resolution 2.6 × 2.6 mm; TA 2:06 min/bed position.

### 2.3. Computed Tomography

CT examinations were performed on a dedicated CT system (Siemens Flash or Siemens Somatom AS, Siemens Healthcare GmbH, Erlangen, Germany) using automated tube current modulation and automated tube voltage selection (CareDose 4 D and CareKV, Siemens Heathcare GmbH). A bodyweight-adapted dosage of iodinated contrast medium was administered intravenously 70 s before the scan. Thoracal images were reconstructed in the lung window setting, using a sharp kernel (B70 s) and a slice thickness of 2 mm.

### 2.4. Image Analysis

The chest imaging datasets of the [^18^F]FDG-PET/MRI examination and CT were analysed on a dedicated OsiriX workstation (Pixmeo, SARL, Bernex, Switzerland) by two experienced radiologists in a random order and in separate sessions, with at least two weeks apart to avoid recognition bias. Any discrepancies between the two readers were resolved in a subsequent consensus reading. In PET/MRI, the VIBE and HASTE sequence were evaluated both independently of one another and considered together. None of the missed lung nodules showed a FDG uptake in the [^18^F]FDG-PET component.

Both readers were informed regarding the primary diagnosis of the patients but remained blind to the results of prior and follow-up imaging and to the patients’ histories.

**Figure 1:**
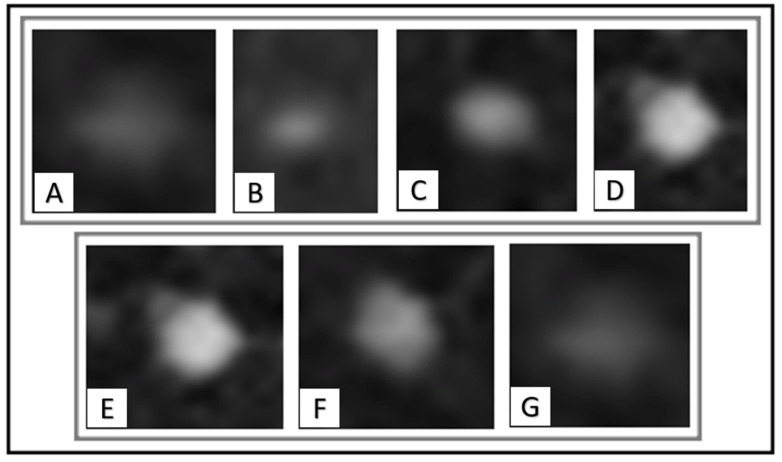
Examples of lung nodule contrast categorization ((**A**): very low contrast; (**B**): low contrast; (**C**): moderate contrast; (**D**): high contrast) and density categorization ((**E**): solid; (**F**): part-solid; (**G**): pure ground glass) at CT.

The quality of all thoracic imaging datasets was evaluated on a four-point Likert scale to differentiate between (1) very poor image quality with major artifacts, (2) poor image quality with moderate artifacts, (3) good image quality with minor artifacts and (4) excellent image quality without any artifacts. The presence and type of artifacts was documented. Lungs were systematically assessed in the same order, dividing the lungs into a (1) right upper, (2) left upper, (3) right lower and (4) left lower quadrant. The amount of lung nodules and the location was noted. Further lung sections were subdivided into a pleural, a subpleural (max. of 1 cm distance to pleura), a central and a hilus region. Nodule size was measured at long axis in mm. Furthermore, nodule contrast was categorized by (1) very low contrast, (2) low contrast, (3) moderate contrast and (4) high contrast. Nodule density was divided into (1) solid, (2) part-solid and (3) pure ground glass (see Figure 1). The nodule shape was classified into (1) round, (2) oval, (3) ellipsoid, (4) lobular, (5) notched and (6) irregular. Initially, a maximum of 10 lung nodules was identified for each patient on baseline imaging. Subsequently, every lung nodule was classified as (1) benign, (2) follow-up needed or (3) malign according, to the actual Fleischner guidelines.

### 2.5. Standard of Reference

**Figure 2:**
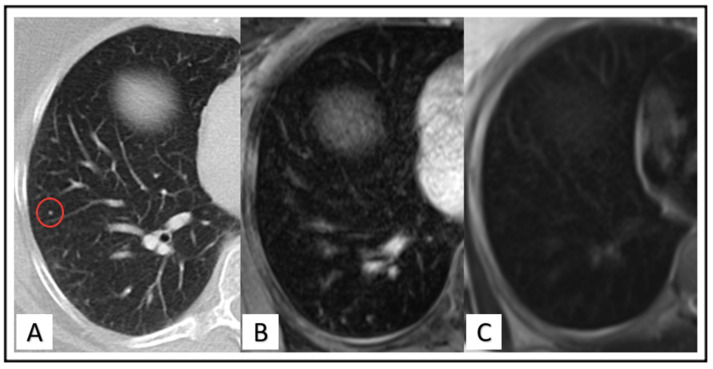
Images of a 46-year-old woman. Four-millimeter lung nodule located in right lower quadrant (red circle) identified on the CT image (**A**) but not recognizable in VIBE (**B**) nor HASTE (**C**) sequence.

**Figure 3:**
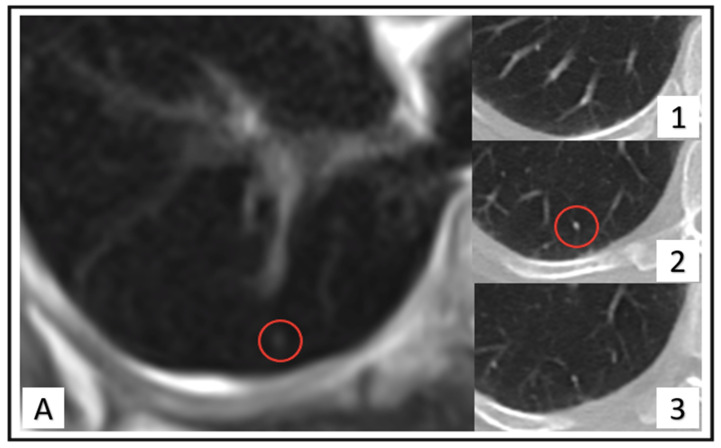
Images of a 62-year-old woman. Three-millimeter lung nodule located in right lower quadrant (red circle) identified at HASTE sequence (**A**) that turned out as vessel in CT (**1**–**3**), continuous structure) due to slice-thickness artifact, evaluated as false-finding.

The reference standard was established in a two-step approach. First, chest CT served as a reference standard compared with PET/MR to determine the number of missed pulmonary nodules and/or false findings on MRI. Each lung nodule found on the initial chest CT dataset but not detected on [^18^F]FDG-PET/MRI has been rated as a missed nodule (see Figure 2). Contrarily, each lung nodule found on [^18^F]FDG-PET/MRI but not definable in chest CT was categorized as a “false-finding” (see Figure 3). The missed lung nodules in PET/MRI were categorized as benign, follow-up needed or malignant, based on CT findings. In a second step, all lesions initially categorized as follow-up needed or malignant were rated again in the follow-up examination. Follow-up imaging includes either chest CT (*n* = 27), MRI (*n* = 93) or both (*n* = 32). The average follow-up interval for chest CT was 6.4 months (range: 2.8 to 27.5 months) and 15.5 month (range: 10 to 29 months) for a whole-body MRI.

New lung nodules at follow-up were not included in the evaluation. Missed lung nodules in MRI, that were rated as benign in initial CT or that need no further follow-up after first follow-up examination were rated as “non-relevant missing”. All missed lung nodules that were rated as malignant in initial CT or that needed further evaluation after first follow-up examination were rated as “relevant missing”. Due to clinical and ethical standards as well as the small size and localization of the evaluated lung nodules, a histological confirmation was not possible.

### 2.6. Statistical Analysis

SPSS Statistics 26 (IBM Inc., Armonk, NY, USA) was used for statistical analysis. Both patient-based and lesion-based data analysis was performed patient-based, and data are presented as mean ± SD. Confidence intervals were calculated at 95%. Using the McNemar test, performance deviation between MRI VIBE and MRI HASTE in lung nodule delineation was analyzed.

## 3. Results

### 3.1. Patient Based Analysis

In initial CT, lung nodules were present in 84/152 (55%) of the women. Out of these 84 patients, 46 (55%) were missed in MRI VIBE, 67 (80%) in MRI HASTE, and 46 (54%) taking MRI VIBE and MRI HASTE into account. While no signs of malignancy were seen in corresponding CT in the majority of the women with missed lung nodules in MRI VIBE (70/84; 83%), a total of 14/84 (17%) of the missed women required further follow-up evaluation based on the CT information. Corresponding results were 25/84 (30%) for MRI HASTE and 14/84 (17%) for MRI HASTE and VIBE (see Table 1A). In women missed by MRI VIBE or MRI VIBE and HASTE, follow-up examinations did not reveal suspicious pulmonary nodules, while one woman missed by MRI HASTE (1%) showed a growing lung metastasis in the follow-up examination (see Table 1B). In total, 3/84 (4%) women of our cohort showed pulmonary metastases. Additionally, one woman in MRI VIBE, two women in MRI HASTE and three women in MRI VIBE and HASTE showed “false positive” findings, meaning there was a suspicious lung finding without any correlation in the CT.

Thus, on a patients-based analysis no relevant missing occurred based on MRI VIBE or MRI VIBE and HASTE information whereas one (1/84; 1%) relevant missing occurred based on MRI HASTE information.

### 3.2. Lesion Based Analysis

A total of 163 lung nodules were present in the initial CT examinations of all women. A total of 96/163 (59%) of lung nodules were not detected in the MRI VIBE datasets and 138/163 (85%) in the MRI HASTE datasets (see Table 2A). Thus, MRI VIBE outperforms MRI HASTE in the detectability of lung nodules (*p* < 0.01).

Out of the 96 missed lung nodules in MRI VIBE, 36 (38%) needed further follow-up evaluation, and 1 (1%) was rated as metastasis based on corresponding CT information. Follow-up examination revealed malignancy for an additional four (4%) lung nodules initially missed in MRI VIBE. In MRI HASTE a total of 62 of the 138 (45%) missed lung nodules needed further follow-up evaluation and 2 (2%) were rated as metastases based on corresponding CT information. Follow-up examination revealed malignancy for an additional eight lung nodules (6%) (see Table 2B).

Thus, on a lesion-based analysis a total, of 4/96 (4%) relevant missing lung nodules were seen in MRI VIBE and at 8/138 (6%) in MRI HASTE.

**Figure 4:**
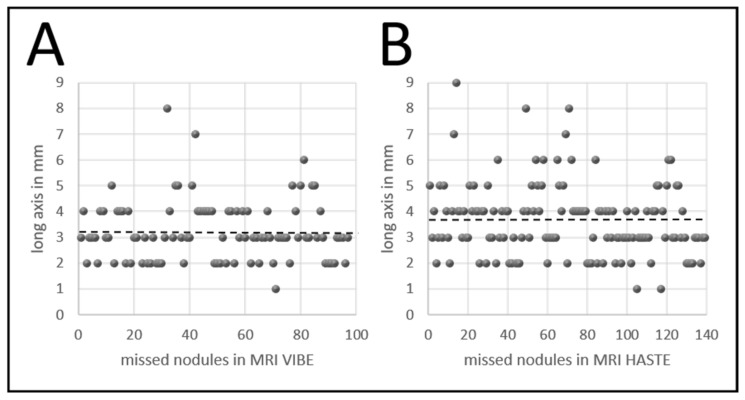
Sizes of missed lung nodules at (**A**) MRI VIBE (*n* = 96) and (**B**) MRI HASTE (*n* = 138) measured on initial CT. Average size (broken line) of nodules was 3.2 mm (range 1–8 mm) in MRI VIBE and 3.6 mm (range 1–9 mm) in MRI HASTE.

The average size of missed lung nodules was 3.2 mm ± 1.2 mm (range of 1–8 mm; 95% CI: 3.4–3.9 mm) at MRI VIBE and 3.6 mm ± 1.4 mm (range of 1–9 mm; 95% CI: 3.0–3.5 mm) at MRI HASTE (see Figure 4). Predominant location of missed lung nodules at MRI VIBE and MRI HASTE was in left lower quadrant (MRI VIBE: 30/48, 63%; MRI HASTE: 43/48, 90%) and close to the hilum (MRI VIBE: 21/21, 100%; MRI HASTE: 20/21, 95%). Lung nodules with very low contrast in CT were not detectable at MRI VIBE and MRI HASTE. Especially ground glass density was unfavorable for MRI detection rate in both sequences (MRI VIBE: 13/20, 65%; MRI HASTE: 18/20, 90%). Round shaped (MRI VIBE: 52/96, 54%; MRI HASTE: 70/138, 51%) and solid density (MRI VIBE: 54/96, 56%; MRI HASTE: 80/138, 58%) was the main characterization of missed lung nodules (see Table 3).

### 3.3. False Findings and Image Quality

Twenty-five additional lung nodules were detected by MRI that were not definable in CT (MRI VIBE: 10/25; MRI HASTE: 14/25; MRI VIBE and HASTE: 1/25). These were characterized as “false findings” generally consistent with a partial volume effect (e.g., vessel summation) after CT correlation. None of the “false findings” turned out to be a “true-finding” in follow-up imaging. A good image quality was achieved in both initial and follow-up imaging (see Table 4).

## 4. Discussion

The improved therapeutic options for breast carcinoma in recent years have led to a special role of primary staging compared to other oncological diseases. While in many carcinomas, the primary therapy strongly depends on the size of the primary tumor and/or locoregional lymph node metastases, in breast cancer the tumor biology is primarily decisive for the primary therapy. The involvement of locoregional lymph nodes is also initially secondary, which is reflected in the oncologically unique term “locally advanced disease”. The aim of image-based primary staging in women with newly-diagnosed breast cancer is thus to primarily exclude distant metastases. At initial diagnosis, up to 10% of women with breast cancer have distant metastases, and especially lung metastases, increases the risk of death in the first year of illness [23]. According to previous studies, 52% to 59% of breast cancer patients show lung nodules at initial staging, underlining our results [24,25]. Thus, detecting and interpreting lung nodules is a relevant part of image-based cancer staging in radiological diagnostics, and lung CT continues to serve as the diagnostic of choice for the detection and classification of pulmonary nodules [15,26,27]. The increasing implementation of [^18^F]FDG-PET/MRI as a primary staging tool for breast cancer patients in a radiation-saving one-stop staging algorithm bears the risk of missing lung nodules, due to a lower sensitivity of the MRI component [11,28]. However, on the other hand, it is questionable if all missed lung nodules lead to a clinical impact or whether they would just lead to additional follow-up imaging to justify benignity. This study serves as one of the largest studies with a homogeneous non-primary metastatic patient collective to evaluate the clinical relevance of missed lung nodules in [^18^F]FDG-PET/MRI staging for women suffering from high-risk breast cancer, contrary to others that include more than one tumor entity with mostly known metastases [15,29,30].

According to the reference standard, no patient with clinically-relevant lung nodules was missed, but there was a small number of undetected clinically-relevant lung nodules in general. Missed lung nodules had a maximum average size of 4 mm and the evaluated data show that most of them are benign (i.e., post-infectious or indurative genesis) concordant to the results of the current literature [15,16]. Here, four (MRI VIBE) or eight (MRI HASTE) lesions were clinically relevant. However, due to the missed clinically-relevant, pulmonary nodules, there were no potential changes in treatment strategy as other clinically-relevant pulmonary nodules had already been diagnosed. In general, a neoadjuvant approach is used to treat women with locally advanced breast cancer, and this approach is followed until there is proof of distant metastases, meaning that since there is a small pulmonal lesion, there will be no switch to a palliative regimen. Thus, pulmonary staging in patients with newly-diagnosed breast cancer can be performed by [^18^F]FDG-PET/MRI. Concordant to previous studies, the detectability of lung lesions greater than 10 mm on PET/MRI was possibly reliable [15,26,31,32]. Taking this together, definite pulmonal metastases dependent on a size larger than 10 mm, as well as an eventual multi-focal appearance, can be accurately detected at initial [^18^F]FDG-PET/MRI staging. Based on our data, there could be missed lung nodules in MRI up to a diameter of 8 mm. Thus, we think that for all breast cancer patients who were initially staged by PET/MRI, a low dose CT of the thorax should be performed after neoadjuvant system therapy has been performed. On the other hand, there is no need to perform a CT in addition to [^18^F]FDG-PET/MRI at initial staging, as this study shows that no lung nodules with direct clinical impact on therapy planning are missed.

Our results confirm the findings of previous studies that MRI VIBE outperforms MRI HASTE for the detection of lung nodules in breast cancer. Smaller lung nodules, especially, were not detected, caused by the higher slice thickness of MRI HASTE [12,29]. Our results support the recommendation of a T1-weighted gradient echo or pulse sequences (i.e., VIBE) for MR-based lung nodule screening with a missing rate of 59% at MRI VIBE and 85% at MRI HASTE [15,33,34,35]. Using newer sequences such as STAR VIBE may reduce the current missing rates [12]. Nonetheless, a relevant portion of lung nodules was missed, especially in the left lower quadrant (MRI VIBE: 63%; MRI HASTE: 90%) and nearly all close to the hilum (MRI VIBE: 100%; MRI HASTE: 95%). Additionally, lung nodules that revealed a very low contrast, as well as a pure ground glass opacity at CT, had an unsurprisingly lower detection rate. Some reasons for that are a low tissue density in the lungs, as well as rapid signal loss at the transition from lung to soft tissue, and artifacts caused by cardiac and respiratory motion, as well as small lung nodule size [12,35]. Concordant to the study of Biondetti et al. (2021), most missed lung nodules had a part-solid to solid density; furthermore, they are predominantly round shaped. Nevertheless, the MRI detectability rate decreases with lower nodule density at CT, as our data show.

Finally using [^18^F]FDG-PET/MRI one-stop whole-body staging algorithm, MRI VIBE sequence represents an essential sequence in lung nodule detection that should be viewed carefully; but, its lower sensitivity in detecting centrally located lung lesions should be kept in mind. 

This study has some limitations. First, CT follow-up was not available for all women. However, under the assumption that lung nodules larger than 10 mm can be detected by MRI more accurately, a relevant growth should be demarcated by MRI [15,26,31,32]. A second limitation is the considerable variability in follow-up intervals. However, an optimal follow-up interval for patients generally suffering from cancer depends on the aggressiveness and doubling time of the underlying tumor histology, and therefore may vary from 6 weeks to 6 months [29,36,37,38]. To counteract this, the planning of follow-up examinations in this study was based on the Fleischner guidelines.

## 5. Conclusions

All women with newly-diagnosed breast cancer and clinically-relevant lung nodules showed positive detection on [^18^F]FDG-PET/MRI initial staging. However, due to the lower sensitivity in detecting lung nodules, a small proportion of clinically-relevant lung nodules are missed. Thus, supplemental low-dose CT of the thorax, after neoadjuvant systemic therapy has been administered, should be considered for backup.

## Figures and Tables

**Table 1:** Patient-based analysis. (**A**) Classification of women with missed lung nodules at MRI VIBE, MRI HASTE and MRI VIBE and HASTE based on initial CT. (**B**) Classification of women with missed lung nodules at MRI VIBE, MRI HASTE and MRI VIBE and HASTE based on initial CT that needed further follow-up imaging.

**(A)**	**Correspond to Initial CT**			
	**Missed Women with Lung Nodules Total**	**Benign Ratings**	**Follow-Up Needed**	**Sign of Malignancy**
**VIBE**	46/84	32/84 (38%)	14/84 (17%)	0/84
**HASTE**	67/84	42/84 (50%)	25/84 (30%)	0/84
**VIBE and HASTE**	45/84	31/84 (37%)	14/84 (17%)	0/84
**(B)**	**Correspond to Follow-Up (MRI/CT)**			
	**Follow-Up Needed Women Total**	**Benign Ratings**	**Further Follow-Up Needed**	**Sign of Malignancy**
**VIBE**	14/84	14/84 (17%)	0/84	0/84
**HASTE**	25/84	24/84 (29%)	0/84	1/84 (1%)
**VIBE and HASTE**	14/84	14/84 (17%)	0/84	0/84

**Table 2:** Lesion-based analysis. (**A**) Classification of lung nodules at MRI VIBE or MRI based on initial CT. (**B**) Classification of lung nodules at MRI VIBE or MRI HASTE based on initial CT that needed further follow-up imaging.

**(A)**	**Correspond to Initial CT**			
	**Missed Nodules Total**	**Benign Ratings**	**Follow-Up Needed**	**Sign of Malignancy**
**VIBE**	96/163	59/96 (62%)	36/96 (38%)	1/96 (1%)
**HASTE**	138/163	74/138 (54%)	62/138 (45%)	2/138 (2%)
**(B)**	**Correspond to Follow-Up (MRI/CT)**			
	**Follow-Up Needed Nodules**	**Benign Ratings**	**Further Follow-Up Needed**	**Sign of Malignancy**
**VIBE**	36/96	92/96 (96%)	0	4/96 (4%)
**HASTE**	62/138	130/138 (94%)	0	8/138 (6%)

**Table 3:** Localization and characteristics of missed lung nodules at MRI VIBE and MRI HASTE sequence corresponding to CT.

	CT Total	VIBE	HASTE
**Localization (Quadrant):** **1-** **right upper** **2-** **left upper** **3-** **right lower** **4-** **left lower**			
	40	23 (58%)	34 (85%)
	23	13 (57%)	17 (74%)
	52	30 (58%)	44 (85%)
	48	30 (63%)	43 (90%)
**Localization (lung tissue):** **1-** **pleural** **2-** **subpleural** **3-** **central** **4-** **hilum**			
	53	20 (38%)	39 (74%)
	55	33 (60%)	47 (85%)
	34	22 (65%)	32 (94%)
	21	21 (100%)	20 (95%)
**Contrast:** **1-** **very low contrast** **2-** **low contrast** **3-** **moderate contrast** **4-** **high contrast**			
	3	3 (100%)	3 (100%)
	27	12 (44%)	24 (89%)
	63	41 (65%)	54 (86%)
	70	40 (57%)	57 (81%)
**Density:** **1-** **solid** **2-** **part-solid** **3-** **pure ground glass**			
	96	54 (56%)	80 (83%)
	47	29 (62%)	40 (85%)
	20	13 (65%)	18 (90%)
**Shape:** **1-** **round** **2-** **oval** **3-** **ellipsoidal** **4-** **lobular** **5-** **notched** **6-** **irregular**			
	80	52 (65%)	70 (88%)
	54	27 (50%)	43 (80%)
	14	9 (64%)	14 (100%)
	1	1 (100%)	1 (100%)
	2	1 (50%)	1 (50%)
	12	6 (50%)	9 (75%)

**Table 4:** Image quality at initial imaging and follow-up (FU) imaging according to a four-point Likert scale (1—very poor image quality to 4—excellent image quality).

	Initial Imaging	FU Imaging
**Chest CT**	4.0 ± 0.3 (CI: 3.9–4.0)	4.0 ± 0.3 (CI: 3.9–4.0)
**MRI VIBE**	3.5 ± 0.7 (CI: 3.4–3.6)	3.4 ± 0.8 (CI: 3.3–3.6)
**MRI HASTE**	3.6 ± 0.6 (CI: 3.5–3.7)	3.4 ± 0.7 (CI: 3.3–3.6)

## Data Availability

The datasets used and/or analyzed during the current study are available from the corresponding author on reasonable request.
